# Relationship between housing and palliative needs among hospitalized older adults

**DOI:** 10.1093/geroni/igaf122.3007

**Published:** 2025-12-31

**Authors:** Abigail Latimer, Lynden Bond

**Affiliations:** University of Kentucky, Lexington, Kentucky, United States; University of Kentucky, Lexington, Kentucky, United States

## Abstract

In 2024, over 146,000 adults aged 55 and older faced homelessness, many for the first time, with one in four living in poor conditions. Rising housing costs and income loss increase worries over losing housing. Many housing insecure older adults live with chronic health conditions and unmet palliative needs. Our objective is to describe the relationship among homelessness, housing conditions, and palliative needs in a sample of hospitalized older adults. We surveyed 139 hospitalized older adults (≥ 55 years, M = 68 ± 9) about housing conditions and health outcomes during a hospital-based point in time count on January 29, 2025. We used the 5-item Integrated Palliative Care Outcome Scale (IPOS) to assess palliative needs. Most were white (86.4%) men (51.8%) living in a home or an apartment (n = 117), with 16 doubled up and two were experiencing homelessness for the first time. About 15% were worried about their housing; 24.4% reported poor housing conditions with at least one problem. Pain was moderate to overwhelming for 68.9%, 35.8% experienced anxiety most or all of the time, and 46.4% felt at least some peace and most had as much information as they wanted regarding their illness (73.9%). While there were no significant associations between symptoms and housing status, those worried about housing reported greater anxiety over their illness (t(133)= 2.70, p= .008), felt less peace (t(134)= 2.83, p=.003), and did not have as much information as they wanted (t(21.24=3.11, p=.005). Housing worries can heighten health-related anxiety and affect how older adults receive information.

